# Maternal mortality linked to COVID-19 in Latin America: Results from a multi-country collaborative database of 447 deaths

**DOI:** 10.1016/j.lana.2022.100269

**Published:** 2022-08

**Authors:** Fabian Maza-Arnedo, Angel Paternina-Caicedo, Claudio G. Sosa, Bremen de Mucio, José Rojas-Suarez, Lale Say, Jenny A. Cresswell, Luis Andrés de Francisco, Suzanne Serruya, Diana Carolina Franco Pulido Lic, Luis Urbina, Erika Saint Hilaire, César V. Munayco, Fabiola Gil, Erick Rousselin, Leonardo Contreras, Allan Stefan, Alvinzy Velásquez Becerra, Evelyn Degraff, Franco Espada, Victor Conde, Gustavo Mery, Víctor Hugo Álvarez Castaño, Aura Liliana Torres Umbarila, Ivy Lorena Talavera Romero, Yeimy Catherine Rodríguez Alfonso, Raquel Lovato Silva, Jakeline Calle, Cynthia Marlene Díaz -Viscensini, Vicente Nicolas Bataglia Frutos, Elodia Vysokolán Laguardia, Haydee Padilla, Alvaro Ciganda, Mercedes Colomar

**Affiliations:** a Grupo de Investigación en Cuidados Intensivos y Obstetricia (GRICIO), Universidad de Cartagena, Colombia; b Universidad del Sinú, Cartagena, Colombia; c Latin American Center for Perinatology, Women's Health, and Reproductive Health (CLAP/WR), Montevideo, Uruguay; d Corporación Universitaria Rafael Núñez, Cartagena, Colombia; e Department of Sexual and Reproductive Health and Research, UNDP-UNFPA-UNICEF-WHO-World Bank Special Programme of Research, Development and Research Training in Human Reproduction (HRP), World Health Organization, Geneva, Switzerland; f Family, Health Promotion and Life Course (FPL), Pan American Health Organization-World Health Organization, United States; g District Health Secretariat of Bogota, Colombia; h PAHO Representation, Dominican Republic; i San Lorenzo de Los Mina Children Maternity Hospital, Santo Domingo, Dominican Republic; j National Epidemiology, Prevention, and Disease Control Center, Ministry of Health, Perú; k PAHO Representation, Perú; l PAHO Representation, Honduras; m Dr Leonardo Martínez Valenzuela Hospital, San Pedro Sula, Honduras; n Alcaldía Mayor de Bogotá, District Health Secretariat of Bogota, Colombia; o Health and Sports Ministry, Bolivia; p PAHO Representation, Bolivia; q Family, Promotion and Life Course, PAHO Representation, Costa Rica; r Ministerio de Salud y Protección Social, Colombia; s PAHO Representation, Colombia; t National Epidemiological Surveillance Directorate, Ecuador; u Ministry of Public Health and Welfare, Asunción, Paraguay; v Clinicas Hospital, Medical Sciences Faculty, Asunción National University, Paraguay; w PAHO Representation, Paraguay; x Clinical and Research Unit (UNICEM), Montevideo, Uruguay

**Keywords:** COVID-19, SARS-CoV-2, Pregnancy, Puerperium, Maternal mortality, Maternal death

## Abstract

**Background:**

This study aimed to describe the clinical characteristics of maternal deaths associated with COVID-19 registered in a collaborative Latin-American multi-country database.

**Methods:**

This was an observational study implemented from March 1st 2020 to November 29th 2021 in eight Latin American countries. Information was based on the Perinatal Information System from the Latin American Center for Perinatology, Women and Reproductive Health. We summarized categorical variables as frequencies and percentages and continuous variables into median with interquartile ranges.

**Findings:**

We identified a total of 447 deaths. The median maternal age was 31 years. 86·4% of women were infected antepartum, with most of the cases (60·3%) detected in the third trimester of pregnancy. The most frequent symptoms at first consultation and admission were dyspnea (73·0%), fever (69·0%), and cough (59·0%). Organ dysfunction was reported in 90·4% of women during admission. A total of 64·8% women were admitted to critical care for a median length of eight days. In most cases, the death occurred during the puerperium, with a median of seven days between delivery and death. Preterm delivery was the most common perinatal complication (76·9%) and 59·9% were low birth weight.

**Interpretation:**

This study describes the characteristics of maternal deaths in a comprehensive multi-country database in Latin America during the COVID-19 pandemic. Barriers faced by Latin American pregnant women to access intensive care services when required were also revealed. Decision-makers should strengthen severity awareness, and referral strategies to avoid potential delays.

**Funding:**

Latin American Center for Perinatology, Women and Reproductive Health.

**Research in context**

**Evidence before this study**

Despite the million infections and deaths associated with COVID-19 worldwide, few maternal deaths have been reported to be related to SARS-CoV-2 infection. We searched PubMed for (“obstetric” OR “maternal”) AND (“death” OR “mortality”) AND (“COVID-19” OR “coronavirus”) from inception to March, 26 2022. The selection criteria were observational studies evaluating maternal mortality associated with COVID-19. We added a filter for female studies. We found 786 articles, of which 53 were observational studies reporting maternal outcomes. The study reporting the most maternal deaths was conducted in Brazil (1031 deaths), followed by a study in Mexico (309 deaths) and South Africa (39 deaths). The lack of published information in the countries participating of this present study along with the increased number of maternal deaths notified by the PAHO member states encouraged the development of this article.

**Added value of this study**

We found 447 maternal deaths associated with COVID-19, with around 90% of all cases with an identified cause of death related to acute respiratory failure after severe COVID-19 infection. We showed that 35% of maternal deaths associated with COVID-19 were not admitted to critical care.

**Implications of all the available evidence**

We found that almost half of maternal deaths associated with COVID-19 got infected during the third trimester. Around half of pregnant women who died were obese, and around a quarter were 35 or older. We found that a significant percentage of maternal deaths in women with COVID-19 were directly related to acute respiratory failure and around a third were not admitted to ICU. Efforts should be directed to increase awareness for early detection of the COVID-19 severity in the pregnant population throughout the region.

Alt-text: Unlabelled box

## Introduction

Since the first outbreak of COVID-19 was reported in December 2019 in Wuhan, China, the disease has spread rapidly worldwide, leading the World Health Organization (WHO) to declare a Public Health Emergency of International Concern on January 30th of 2020.^1^^,^^2^ As of December 12th of 2021, more than 269 million infections and 5·3 million deaths had been reported for COVID-19 worldwide.^3^

Pregnant and postpartum women are generally more susceptible to developing severe viral infections due to the physiological adaptations occurring during pregnancy.^4^ In prior coronavirus epidemics of severe acute respiratory distress syndrome virus and Middle East respiratory syndrome virus, pregnant women had higher case fatality rates and more complications than nonpregnant women.^5^

Based on data from the first five months of the COVID-19 pandemic, some reports suggested that pregnant women were not at a greater risk of significant adverse outcomes in SARS-CoV-2 infections. In addition, maternal mortality rates due to SARS-CoV-2 appear to have, at least initially, remained in line with pre-pandemic levels with the disease course being primarily benign.^6^, ^7^, ^8^, ^9^, ^10^ Contrary to those first studies, later analysis revealed that infected pregnant women were more likely to be admitted to critical care, receive invasive ventilation, extracorporeal membrane oxygenation treatment and died compared with non-pregnant women of reproductive age.^11^, ^12^, ^13^, ^14^ Currently of the few studies coming from low and middle-income countries that compare maternal mortality levels and trends, pre-and during, the COVID-19 pandemic increased maternal deaths were found.^15^, ^16^, ^17^ There are two hypothetical pathways by which maternal mortality levels might change: either due to the aggravation of SARS-CoV-2 infection due to the pregnant state or due to interruptions in access to maternity services.

Beginning in late March 2021, several countries in the Americas reported a considerable number of COVID-19-related maternal deaths. A partial snapshot of the situation in Latin America (LAC) as of June 15th of 2020 showed more than 100 maternal deaths related to COVID-19 out of a total of 2291 pregnant women who tested positive for SARS-CoV-2 from six LAC countries.^18^

Identifying features that might lead to death is essential in pregnant and puerperium women with COVID-19. Therefore, this study aims to describe and analyze the clinical and epidemiological characteristics of maternal deaths associated with COVID-19 registered in a collaborative multicountry database from Latin America.

## Methods

### Design and settings

This was an observational, descriptive study based on the Perinatal Information System (SIP for its Spanish acronym). The SIP is a perinatal medical record developed by the Latin American Center for Perinatology, Women's Health, and Reproductive Health (CLAP/WR). The system stores information on pregnancy, delivery, and neonatal outcomes in Latin America, shared by ministries or health institutions. The SIP was initially designed by the Pan American Health Organization (PAHO) to monitor clinical care and epidemiological data, improving the quality of care for mothers and newborns.

Due to the pandemic, CLAP/WR developed a specific SIP form to monitor the burden of COVID-19 infection during pregnancy, birth, and postpartum (SIP COVID-19), which can also be used for other acute respiratory infections of interest to public health.

We included maternal deaths linked to COVID-19 diagnosed through PCR testing officially reported by ministries of health and included in their surveillance report, from March 1st of 2020 to November 29th of 2021 in Latin America.

This study was exempt from ethical approval by the PAHO Ethical Review Committee. The authors followed all relevant guidelines and ethical regulations for this study. This manuscript was reported according to the STROBE guidelines for observational studies.

### Data collection procedure

The SIP COVID-19 records demographic data, including epidemiologic history (maternal and gestational trimester at notification date, educational level, obstetric history, and mode of delivery). The SIP COVID-19 form also includes relevant comorbidities (hypertension, diabetes, obesity, deficiency anaemia, including iron anaemia and other causes of deficiency anaemia, tuberculosis, immunocompromised status, including Human Immunodeficiency Virus). Additionally, this form records clinical data, PCR test results, clinical symptoms and signs at first consultation and admission, laboratory tests, and treatments (i.e., antivirals, steroids, convalescent plasma, mechanical ventilation, vasoactive drugs). Additionally, the SIP COVID-19 gathers the clinical evolution, obstetric complications (hypertensive disorder, antepartum and postpartum haemorrhage, and maternal sepsis), perinatal events (including oligohydramnios, intrauterine growth restriction, and stillbirth), and pregnancy outcomes (abortion and preterm delivery). Finally, the form extracts information until the end of the hospital stay (recording if the patient was transferred to an intensive care unit (ICU) and/or the date of maternal death).

We identified two scenarios for data collection. First, the maternal network of sentinel sites through which CLAP/WR implements collaborative studies. This network includes sentinel sites from Dominican Republic, Honduras, and Bolivia. CLAP/WR performs regular surveillance in these sentinel hospitals to track pregnant women who meet the inclusion criteria for this study. The data collection started identifying maternal death cases with a positive PCR test. Subsequently, CLAP/WR contacted the PAHO country office to make sure that the Ministry of Health officially acknowledged the event, and if so, the case became part of the database hosted in CLAP/WR.

The second identification mechanism was through monthly PAHO epidemiological updates based on information shared by International Health Regulations National Focal Points or published on Ministries of Health, Health Agencies, or similar. The study coordination team tracked the number of cases in epidemiological updates by country. Then they asked the PAHO office to contact national authorities and asked them to contribute by coordinating the filling of a COVID-19 SIP form along with the heads of the healthcare centre where the death occurred (see *Appendix* 2). These forms were digitally sent to the PAHO country office or CLAP/WR and entered into the database. At all times, the staff in PAHO maintained strict confidentiality regarding any data that could identify maternal deaths.

One of the analysis limitations is the lack of complete data from some countries. Considering that the data collection took place during the pandemic, some centres did not have enough staff to find requested medical records or lab exams undertaken by women. For this reason, information related to some of the variables in the COVID-19 form may be missing.

### Data analysis

Forms were uploaded without personal identification data, and the report was presented as pooled data with the overall number of cases for each country. We summarized categorical variables as frequencies with percentages and continuous variables into median with interquartile ranges (IQR). We had a substantial amount of missing data for most variables. We report complete data and its denominator for each variable.

We performed all statistical analyses using R Statistical Software (version 4.1.0; R Foundation for Statistical Computing, Vienna, Austria).

### Role of the funding source

This work received funding from the Latin American Center for Perinatology, Women and Reproductive Health (CLAP/WR), PAHO/WHO. The funder was not involved in any methodological decision and was not involved in the submission of the manuscript for publication.

## Results

Ministries of Health from nine Latin American countries joined the study. One country was excluded because they officially acknowledged fewer maternal deaths than reported to the collaborative base. We kept information from eight countries, which formally reported 693 COVID-19 maternal deaths between January 2020 and November 2021. Data was collected for 447 maternal deaths. All the pregnant and postpartum women included had a positive SARS-CoV-2 test, irrespective of the cause of death.

The country that reported the largest number of deaths to this database was Honduras (*n* = 126), followed by Paraguay (*n* = 86), Colombia (*n* = 84), Ecuador (*n* = 55), Perú (*n* = 34), Dominican Republic (*n* = 30), Bolivia (*n* = 21), and Costa Rica (*n* = 11). We obtained information from more than 90% of the maternal deaths linked to COVID-19 officially reported by Costa Rica, Ecuador, Honduras, and Paraguay, 66·7% from the Dominican Republic's cases, 43·5% for Colombia, 41·2% from those that occurred in Bolivia, and 17·9% for Peru.

**Figure 1:**
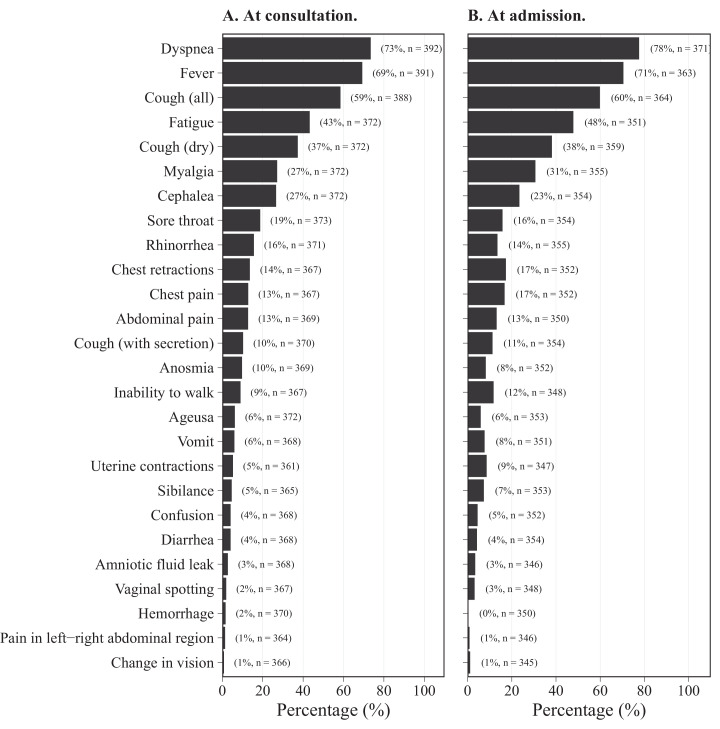
The onset of symptoms at first consultation (**Panel A**) or hospital admission (**Panel B**) for maternal deaths with COVID-19.

The median maternal age at death was 31 (IQR, 26-36), while 25·3% of deaths occurred in women aged 35 or older. Most women (92·2%) had a nonwhite ethnicity and almost half of them completed high school. The median number of previous pregnancies was 2 (IQR, 1-3; *n* = 393), and 23·4% (88/376) were nulliparous. Eight of the dead women were health workers. Almost half of the women from the database had a body mass index that categorized them as obese; 9·2% (35/380) had a history of diabetes mellitus and 8·4% (32/380) had a history of chronic hypertension; 1·6% (6/371) suffered from human immunodeficiency virus infection and 1·3% (5/377) had a history of tuberculosis (Table 1). There were no relevant differences between countries with these characteristics. (*Appendix* 2) At first consultation and admission, the most frequent symptoms were dyspnea (73·0%), fever (69·0%), and cough (59·0%) (Figure 1).

**Table 1:** Characteristics of pregnant and puerperium dead women with COVID-19 from Latin America.

Characteristics	Value	Available data
	*n* (*%*)	*n*
**Maternal age**		431
<20	24 (5·6%)	
20-35	298 (69·1%)	
> 35	109 (25·3%)	
**Ethnicity**		282
White	22 (7·8%)	
Nonwhite (Afro, black, indigenous, mestiza, other)	260 (92·2%)	
**Education**		365
No studies	10 (2·7%)	
Incomplete high school	111 (30·4%)	
Complete high school	183 (50·1%)	
University	61 (16·7%)	
**Obstetric history**		376
Nulliparous	88 (23·4%)	
1-2 previous deliveries	202 (53·7%)	
Multiparous	86 (22·9%)	
Cesarean sections	155 (44·0%)	352
**Mode of delivery**		282
Vaginal delivery	44 (15·6%)	
Cesarean section	238 (84·4%)	
**Medical history**		
Hypertensive disorders:		
Chronic Hypertension	32 (8·4%)	380
Preeclampsia	28 (7·4%)	378
Eclampsia	6 (1·6%)	376
Obesity	83 (49·4%)	168
Diabetes mellitus	35 (9·2%)	380
Tuberculosis	5 (1·3%)	377
HIV	6 (1·6%)	371
Asthma	25 (6·7%)	373
Smoking	1 (0·3%)	367
Recreational drug use	5 (1·4%)	369
Alcohol use	4 (1·1%)	368

**Figure 2:**
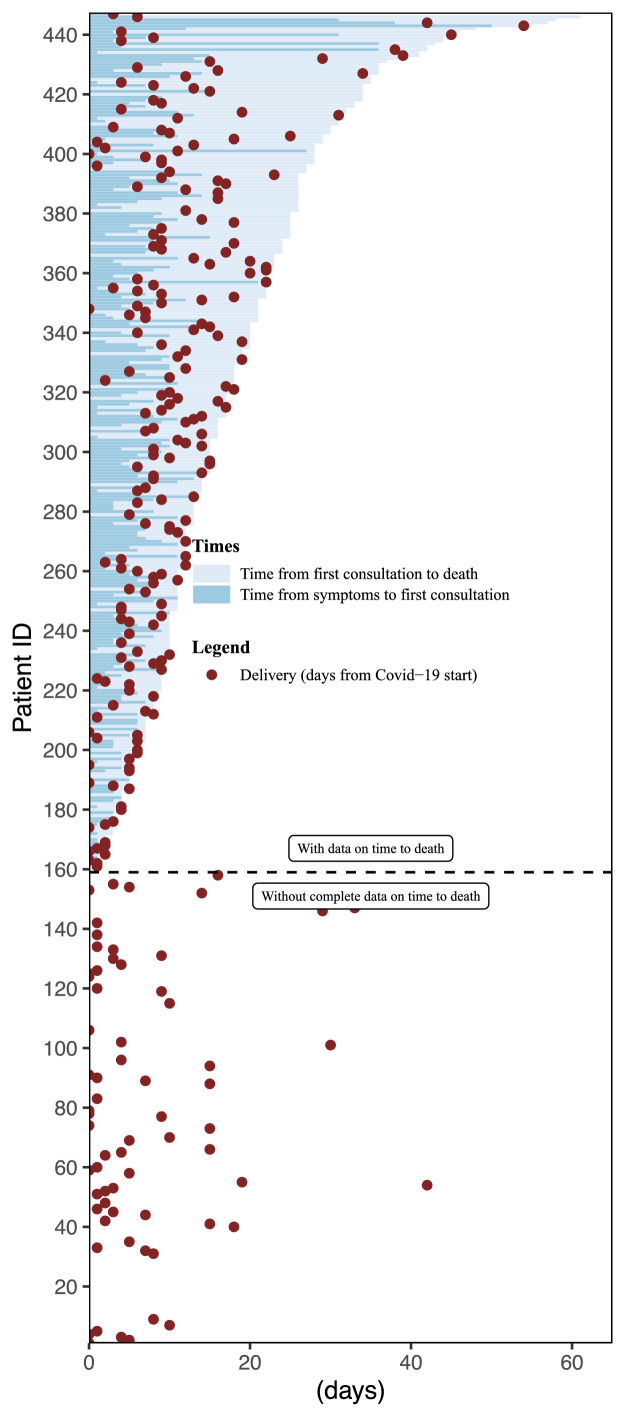
Time from symptoms onset to first consultation, delivery and death in pregnant and postpartum women with COVID-19 in Latin America (n = 447).

Among all maternal deaths, 185 (52·1%) were recorded in the third trimester. At the moment of infection, the median gestational age was 31 weeks (IQR, 26-35), with few cases of maternal deaths occurring during the first trimester (14 cases [3·9%]). 19·0% (85/446) of the women died in ≤ 48 h after delivery and 34·3% (153/446) died more than seven days after delivery. 33·8% (151/446) of patients lack information about the time between delivery and maternal death. Among patients with information, those infected during pregnancy, the median time between hospital admission and delivery was two days (IQR, 0-5). The median time between the onset of symptoms and delivery in the entire sample was seven days (IQR, 2-12), and between the onset of symptoms and death was 14 days (IQR, 8-25) (Figure 2). Death in 99·7% (344/345) of cases occurred during the puerperium, with a median of seven days (IQR, 1-15) between delivery and death.

Of the fatalities, antibiotics were prescribed in 76·7% (266/347) of women, tocolytics in 6·6% (19/287) women, ivermectin in 4·8% (14/293), antimalarials in 3·4% (10/292), and antiepileptic drugs in 1·2% (3/253), Other interventions, such as steroids, convalescent plasma, or interleukin antagonists, were not reported.

Table 2 shows the prevalence of obstetrical complications for maternal deaths with COVID-19. Around 19·7% (71/360) of women had hypertensive disorders during pregnancy. Among clinical and laboratory data, the oxygen saturation had a median of 90·0% (IQR 81-95; *n* = 199), with levels below 95·0% in 62·0% (116/199) of cases reported. Among laboratory variables, the PaO_2_/FiO_2_ was reported <200 in 25·1% (66/263) of cases, with lower values reported in maternal mortality cases during the second and third trimesters. Thrombocytopenia was infrequent in our population (with a median of 237 × 10^9^ platelets/L [IQR, 176-321]), as well as leukocytosis (the median was 8,700 cells/mm^2^ [IQR, 1,429-14,700]). Other median values with an interquartile range of laboratory values by gestational trimester during a hospital stay are shown in Supplementary Figure S1.

**Table 2:** Onset timing, complications, and complications of deaths in pregnancy and puerperium.

Parameter	Value	Available data
	*n* (*%*)	*n*
**Symptom's onset**		355
1st trimester	14 (3·9%)	
2nd trimester	108 (30·4%)	
3rd trimester	185 (52·1%)	
Puerperium	48 (13·5%)	
**Pregnancy complications**		
Hypertensive disorders	71 (19·7%)	360
Diabetes mellitus	44 (12%)	366
Hemorrhagic disorders	45 (12·6%)	358
Thrombotic events	13 (3·6%)	358
Pneumonia/Abnormal chest X ray	210 (83·7%)	251
**Level of care**		
Critical care	219 (64·8%)	338
Minimum care (do not require organ support)	119 (55·1%)	216
**Perinatal events**		
Abortions	21 (8·3%)	253
Stillbirth	76 (24·6%)	309
Preterm birth	214 (78·4%)	273
Low birth weight (<2,500 grs)	112 (59·9%)	187
**Obstetric complications**		
Intrauterine growth restriction	4 (1·39·0%)	357
Premature rupture of membranes	5 (1·39·0%)	359
Obstructed labor	5 (1·4%)	360
Oligohydramnios	11 (3·1%)	358
Polyhydramnios	2 (0·6%)	359
Non-reassuring fetal status	62 (17·1%)	363
Cholestasis	2 (0·6%)	358
Chorioamnionitis	2 (0·6%)	355
Hyperemesis gravidarum	3 (0·8%)	358
Amniotic fluid embolism	0 (0·0%)	358
**Causes of death**		447
**Direct causes**		
Hypertensive disorders (HELLP)	3 (0·7%)	
Postpartum hemorrhage	4 (0·9%)	
Obstetric sepsis	4 (0·9%)	
Embolism	1 (0·2%)	
**Indirect causes**		
Acute respiratory failure associated with severe COVID-19 infection	315 (70·5%)	
Other causes of death	25 (5·6%)	
**Undetermined**	95 (21·3%)	

**Figure 3:**
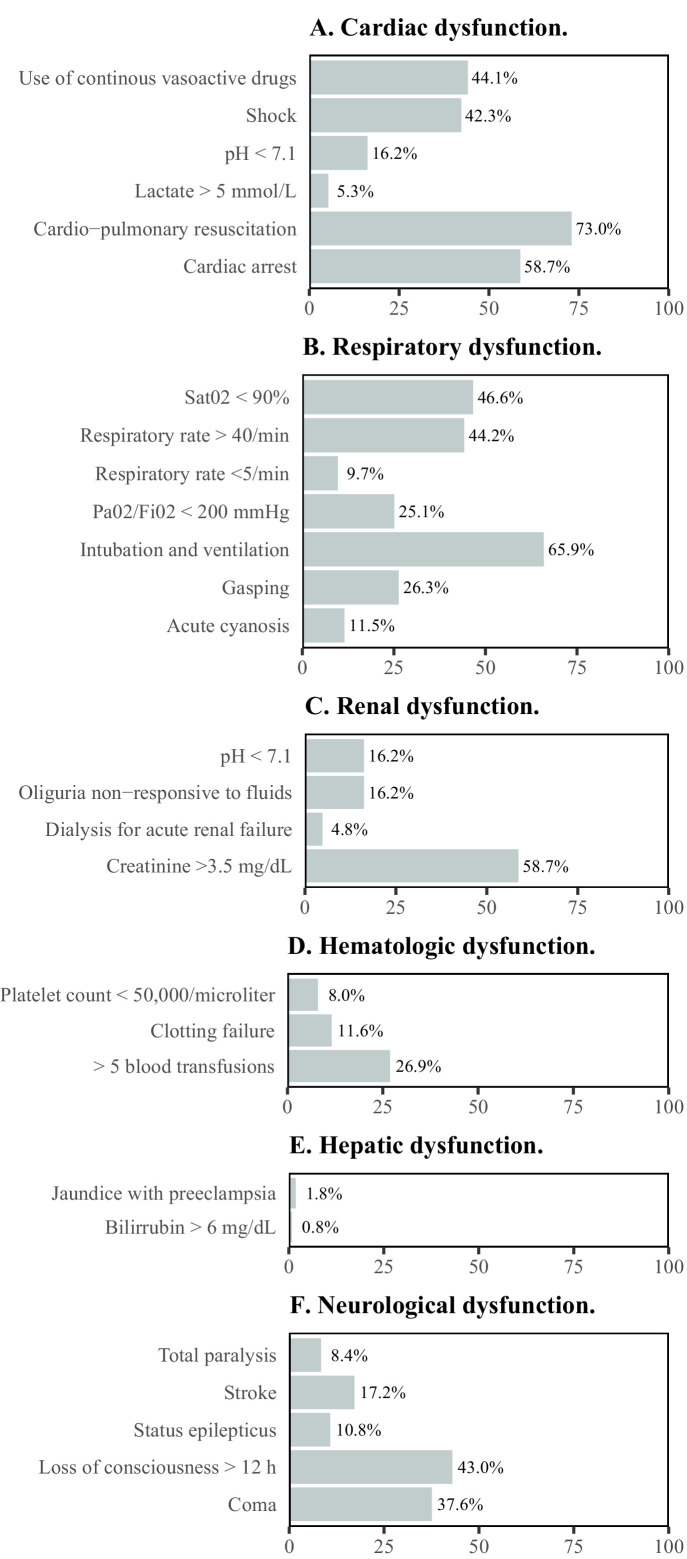
Organ dysfunction for maternal deaths with COVID-19 in Latin America, according to the WHO classification.

Organ dysfunction was reported in 90·4% (322/356) of women during the hospital stay, according to the WHO Maternal Near Miss criteria. Based on the clinical criteria, 67·2% (216/321) of women suffered from cardiac dysfunction, 55·2% (164/297) experienced respiratory dysfunction, 23·8% (69/290) had neurologic dysfunction, 16·2% (48/296) renal dysfunction, 11·6% (33/285) hematologic dysfunction and 1·8% (5/279) hepatic dysfunction. Around half of the women fulfilled at least one laboratory-based criterion for MNM, while ¾ fulfilled one management-based criterion prior to death and 50·2% (160/319) received cardiopulmonary resuscitation (Figure 3).

Preterm delivery was the most common perinatal complication (76·9%, 214/278). At birth, the median gestational age was 33 weeks (IQR, 30·0-36·0; *n* = 273) and 59·9% (112/187) were low birth weight. Median APGAR at five minutes was eight (IQR, 5-9; *n* = 185). Additionally, 8·3% (21/253) of pregnancies ended in abortion (Table 2).

Considering the characteristic of the care received by the women who died with COVID-19 in participating countries, 64·8% (219/338) were admitted to critical care, with a median length of stay of eight days (IQR, 3·7-16·0; *n* = 172). A total of 30·4% (108/355) were infected during the second trimester, 52·1% (185/355) in the third trimester, and 13·5% (48/355) started symptoms during puerperium (Table 2). The most frequent cause of death was respiratory failure associated with severe COVID-19 infection (70·5%; *n* = 315/447).

## Discussion

We present the first multicountry description of pregnant and postpartum women characteristics that died with COVID-19 in Latin America. Our study adds to the body of knowledge on the path to death in maternal patients in the region.

In the present study, obesity, hypertensive disorders, and diabetes were the most common comorbidities present among pregnant women who died with COVID - 19. Almost all causes of death in our study were linked to acute respiratory distress syndrome (ARDS). The most common organ dysfunctions in our population were cardiovascular and respiratory. Most of our patients presented severe hypoxemia (oxygen saturation ≤ 90·0%) and severe tachypnea, with 62·0% showing oxygen levels below 95·0%. Additionally, more than half of renal dysfunction was related to a significant increase in creatinine levels. Around a third of maternal deaths did not have critical care admission or respiratory support, potentially impairing their survival risk. The lack of critical care in about one out of three patients might reflect barriers to access to intensive care faced by pregnant women, for example, the limited number of beds or the administrative referral process. Low admission rates may also be related to overwhelmed healthcare systems throughout the region and the lack of critical care beds in these countries. In this database, we accounted for eight maternal deaths from health workers, and only one of them was not admitted to an ICU before dying.

Comorbidities such as asthma, obesity, diabetes, and hypertensive disorders are considered significant risk factors to develop severe complications of COVID-19 during pregnancy.^11^^,^^12^^,^^19^^,^^20^ Comparing our figures with other studies that reported comorbidities for maternal deaths associated with COVID-19, the largest difference was seen in the rate of obesity. We reported the highest rate (49·4%). This figure was based on 168 cases and might be affected by the large amount of missing data. A large study analyzing 197 maternal deaths in Mexico described a rate of 16·4%, another study including 124 pregnant women who died with COVID-19 in Brazil reported almost 21·3%, and another study including seven maternal deaths in Iran reported 14·3%. A study in India including 34 maternal deaths did not report their obesity rate.^21^, ^22^, ^23^, ^24^

In addition to obesity, there were other important comorbidities. The reported diabetes rate (gestational or previous) in Brazil was almost three times the rate we found (12%).^22^ While Mexico and Iran were the closest to our figure with 11·3% and 14·3%, respectively, India reported the lowest rate, with 5·9%.^21^^,^^23^^,^^24^ The asthma rate in our study (6·7%) was close to what was reported in Brazil (9·3%) but significantly higher than what was reported in the Mexican study (2·6%).^22^^,^^23^ Finally, chronic hypertension was 2·9% in the Indian study. While the Mexican study reported 7·7% similarly to our finding (8·4%).^21^^,^^23^ Differences found between comorbidity rates might be explained by small sample sizes and data limitations.

Our study strengthens evidence showing the harmful effect of hypertensive disorders on pregnancy in patients with COVID-19. According to a meta-analysis, COVID-19 is associated with a considerable risk of preeclampsia, preterm birth, and low birth weight.^25^ Furthermore, our analysis showed that 19·7% of all deaths were complicated by hypertensive disorders of pregnancy. While only 3·0% of low-risk pregnancies are normally complicated by preeclampsia in the region of the Americas and 4·6% globally.^26^

In Latin America, we report a median age for maternal deaths associated with COVID-19 at 31 years (IQR, 26-36), consistent with the Brazilian study.^22^ Our data, with a quarter of deaths occurring in advanced maternal ages, highlights the importance of increasing awareness among decision-makers of the risk of death in obstetric women infected with COVID-19 at older ages as reported in other studies.^15^^,^^19^^,^^21^^,^^24^^,^^27^^,^^28^ The present study found that fever, cough, and dyspnea were the most frequently reported symptoms in pregnant women with COVID-19 at consultation and at the time of hospital admission, consistent with previous publications.^12^^,^^13^^,^^21^^,^^29^, ^30^, ^31^, ^32^ A cohort study revealed that severe pregnancy and neonatal complication rates were highest in women if fever and shortness of breath were present.^8^ Consistent with our data, those clinical manifestations were more frequent among women included in this study than among less severely ill pregnant women.^12^^,^^20^ Most women in this study were infected during the third trimester of pregnancy and similar to other reports, the timing for most maternal deaths was during puerperium.^15^^,^^22^^,^^24^^,^^33^^,^^34^

Our study rarely reported organ dysfunction based on laboratory tests at any point during hospital stays, possibly due to lack of information or difficulties during data collection. Beyond this, the presence of early signs of respiratory failure (decrease in oxygen saturation and oxygenation index) and hemodynamic instability with vasoactive requirements are well-known risk factors for mortality following COVID-19 infection.^35^ However, the limits and warning signs in the obstetric population differ from those of nonpregnant patients.^36^ Therefore, a healthcare team composed of intensivists and OB-GYNs following jointly defined protocols is crucial to prevent maternal mortality and morbidity due to COVID-19 among pregnant women.

In this study, women from LACs who died with COVID-19 had a high frequency of preterm birth because of their coronavirus disease, similar to other studies that included pregnant women with COVID-19.^8^^,^^14^^,^^25^^,^^27^^,^^28^^,^^34^^,^^37^ That was the most common perinatal complication and stillbirth was described in one-quarter of cases. These data reflect the complex maternal-fetal interaction during severe COVID-19 cases. Moreover, a non-reassuring fetal status was found in 20·0% of patients, confirming this challenging situation. Fetal monitoring in a critical condition or severe high-risk patients requires a multidisciplinary team, including a maternal-fetal specialist, anesthesiologist, and a critical care physician, among others. This higher level of care is infrequent in many Latin American facilities, leading to elevated rates of perinatal and maternal morbidity and mortality. Early detection, and increased awareness of severity, leading to appropriate and timely referral systems, are necessary to overcome this lack in maternity care.

The aggregation of these diseases, alongside a background of social and economic disparity, exacerbates the adverse effects of each separate disease, which has caused some authors to call COVID-19 syndemic.^38^ In addition, several studies in a variety of contexts, including Latin America, reported ethnic inequality-related factors on COVID-19 mortality or severe morbidity, nonwhite being a risk factor.^12^^,^^39^, ^40^, ^41^ This leads us to conclude that if maternal death is considered the “tip of the iceberg” on disparities, then the population from this study, pregnant women who died with COVID-19, could be considered a window into a disadvantaged population with many overlapping contributors.

Our findings put into evidence how already strained healthcare systems in this pandemic setting and overcrowded health facilities had a significant impact on maternal mortality. In those scenarios, the WHO three-delays framework gains relevance for understanding the gaps in access to adequate management of obstetric emergencies, delays in seeking appropriate medical help for an obstetric emergency, reaching an appropriate obstetric facility, or receiving adequate care when a facility is reached.^42^

Reports from other studies revealed similar findings among pregnant women who died. In Brazil, a study reported that the lack of access to an ICU varied from 0·0% to 50·0%, and the lack of invasive ventilation ranged from 0·0% to 51·5%, while another study from the same country revealed that 22·6% did not access an ICU.^22^^,^^43^ A study from Mexico reported seven maternal deaths, with only two women admitted to critical care.^15^ Similarly, a cohort study implemented in 18 countries reported that deaths occurring in women with COVID-19 diagnosis were concentrated in institutions from less developed regions, implying that when comprehensive ICU services are not fully available COVID-19 in pregnancy can be lethal.^8^

In low and middle-income countries pregnant women with COVID-19 have eight times greater risk of dying compared to those in high-income countries. The maternal mortality case fatality rate, due to COVID-19, in the participating countries had a median of 1·7 IQR (0·8 - 3·8).^44^ This difference in risk is directly related to the lack of adequate infrastructure, access to healthcare services, lack of knowledge of the critical illness during pregnancy, and the impact of the COVID-19 pandemic on healthcare services across regions.^19^ COVID-19 has increased the maternal mortality rate, generating a setback in the region's achievements in the last decade.^45^ Moreover, COVID-19 and its impacts are taking a toll on the health of women and newborns, as mothers continue to face disruptions in prenatal care and delivery. In 2020, the Latin American mortality ratio was 88 deaths per 100,000 live births, up from 83 deaths per 100,000 live births in 2019.^46^

In addition, information regarding case fatality rates among women of reproductive age is not consistently available, impairing equitable responsiveness during pandemic recovery.^47^ Our study suggests Latin American women have difficulty accessing higher levels of care and this occurred also among the general population. Consistently, a study in Mexico including the adult population described hospital overcrowding and reported that 45·6% of dead patients were not admitted to ICU.^48^ These figures reflect how the pandemic also impacted the general population in settings that were not prepared for a highly demanding sanitary situation. Urgent measures are needed to address access to healthcare services that target pregnant women, prioritizing well-defined intensive care criteria for pregnant and postpartum women, as well as the general population.

By December 12, 2021, more than 8 billion doses of the COVID-19 vaccine had been administered worldwide.^49^ Nevertheless, this global figure hides significant inequalities between continents and income groups since vaccine doses have so far been distributed unevenly.^50^^,^^51^ For instance, during the first semester of 2021, Latin America had a lower vaccination coverage compared to North American or European countries.^49^^,^^52^ Countries like Guatemala, Honduras, Nicaragua, Jamaica, Haiti, and Venezuela did not even reach vaccination coverage of 1% by June 2021.^49^^,^^52^ Therefore, due to the vaccine scarcity, countries have designed vaccination plans prioritizing the most vulnerable population. In July 2021, PAHO's Technical Advisory Group (TAG) on Vaccine-Preventable Diseases recommended prioritizing vaccination among pregnant women from the Americas,^53^ while WHO's Strategic Advisory Group of Experts on Immunization (SAGE) placed pregnant women in Stage II of the epidemiological scenarios, in particular women of higher age, and those with comorbidities.^54^ Also, the CDC recommends COVID-19 vaccination for everyone aged five years and older, including pregnant women, lactating, or who might become pregnant in the future.^55^ Despite recommendations for vaccination, uptake of COVID-19 vaccination by pregnant women has been lower than for the nonpregnant population.^56^ Moreover, according to a recent update received by SAGE on ICU admissions of pregnant women and impacts of COVID-19 on pregnancy, vaccination levels and plans to improve confidence in, and uptake of, vaccines among this group indicate that low rates of vaccination are a major risk factor among pregnant women.^57^

The present study has several limitations. One of the limitations is a large number of missing data on variables from the clinical record. Considering that the COVID-19 pandemic stressed healthcare systems to their maximum capacity, mainly in low resource settings, we expected this limitation due to the shortage of human resources. Unfortunately, missing data could affect estimates for each factor. We also did not record dates of laboratory reports, so inference on time to event is limited. Additionally, the form did not record economic information and place of residence to contribute to the discussion related to vulnerability. Another limitation is the incomplete representation of maternal deaths linked to COVID-19 from participating countries due to operational delays. Moreover, we did not get information from all countries in Latin America, particularly Brazil and Mexico with the greatest contribution to maternal death in the region, which might have produced an imbalance if differences existed. Finally, due to the study design and because we did not include different populations, such as; dead pregnant women COVID-19 free; dead not-pregnant women with COVID-19; or pregnant women who survived COVID-19; we could not calculate differences in risks factors associated with COVID-19 and pregnancy.

As the main strength, this study is the first multicounty registry of maternal deaths linked to COVID-19 including Latin-American countries that allows characterizing maternal deaths in the region. In addition, is the largest study reporting frequency of comorbidities and onset of symptoms related with COVID-19 in the Latin-American region. These analyses could provide relevant information on the behaviour of COVID-19 in the LAC obstetric population.

In conclusion, the present study provides valuable insight into the presentation of maternal mortality associated with COVID-19 among LAC women. Additionally, we found healthcare barriers faced by LAC pregnant women to access intensive care services. Decision-makers should strengthen severity awareness, and referral strategies to avoid potential delays in the care of obstetric patients. We further recommend increasing the capacity of attending to severe maternal patients across LAC countries, especially defining protocols and expanding critical care beds according to the Latin-American country's needs.

## Contributors

Fabian Maza-Arnedo, wrote the original draft and search literature. Angel Paternina-Caicedo performed the data analysis, literature search and writing. Claudio G. Sosa; Bremen de Mucio; José Rojas-Suarez and Mercedes Colomar designed the study, interpreted the data, and contributed to the writing. Lale Say, Jenny A Cresswell, Luis Andrés de Francisco, Suzanne Serruya reviewed the manuscript and contributed to the writing. Alvaro Ciganda validated the data and performed the quality control. Erika Saint Hilaire; Jakeline Calle; Vicente Nicolas Bataglia Frutos; Victor Conde; Raquel Lovato Silva; Leonardo Contreras; Allan Stefan; Carlos Ochoa, Elodia Vysokolán Laguardia collected the data and reviewed the manuscript. César V. Munayco, Fabiola Gil, Erick Rousselin, Evelyn Degraff, Franco Espada, Gustavo Mery, Julián Alfredo Fernández Niño, Víctor Hugo Álvarez Castaño, Leonor Guavita Cita, Aura Liliana Torres Umbarila, Diana Carolina Franco Pulido, Alvinzy Velásquez Becerra, Ivy Lorena Talavera Romero, Yeimy Catherine Rodríguez Alfonso, Rosalinda Hernandez, Cynthia Marlene Díaz -Viscensini, Haydee Padilla, Dolores Altagracia Rodríguez Lappot, Maria Santos, Luis Urbina; supervised the field work and reviewed the manuscript.

## Data sharing statement

Data supporting this study's findings are available upon reasonable request to the corresponding author.

## Disclaimer

Authors as staff members of the Pan American and World Health Organization are responsible for their views expressed in this publication and do not necessarily represent the decisions or policies of the Pan American and World Health Organization or the UNDP-UNFPA-UNICEF-WHO-World Bank Special Programme of Research, Development and Research Training in Human Reproduction (HRP).

## Funding

This work received a grant from the Latin American Center for Perinatology, Women and Reproductive Health (CLAP/WR), PAHO/WHO. The funder did not influence the study design, data collection, data analysis, or the decision to submit the manuscript for publication.

## Declaration of interests

None.

## Appendix Supplementary materials

Image, application 1

Image, application 2

Image, application 3

Image, application 4

## References

[1] World Health Organization (WHO) (2020).

[2] World Health Organization (WHO) (2020).

[3] Johns Hopkins University & Medicine (2021).

[4] Dotters-Katz S., Hughes B.L., Miller E., The Society for Maternal-Fetal Medicine (SMFM) (2020).

[5] Schwartz D.A., Graham A.L. (2020). Potential maternal and infant outcomes from Coronavirus 2019-nCoV (SARS-CoV-2) infecting pregnant women: lessons from SARS, MERS, and other human Coronavirus infections. Viruses.

[6] Vizheh M., Muhidin S., Aghajani F. (2021). Characteristics and outcomes of COVID-19 pneumonia in pregnancy compared with infected nonpregnant women. Int J Gynecol Obstet.

[7] Karimi L., Makvandi S., Vahedian-Azimi A., Sathyapalan T., Sahebkar A. (2021). Effect of COVID-19 on mortality of pregnant and postpartum women: a systematic review and meta-analysis. J Pregnancy Hindawi Ltd.

[8] Villar J., Ariff S., Gunier R.B. (2021). Maternal and neonatal morbidity and mortality among pregnant women with and without COVID-19 infection: the INTERCOVID multinational cohort study. JAMA Pediatr.

[9] Chen L., Li Q., Zheng D. (2020). Clinical characteristics of pregnant women with Covid-19 in Wuhan, China. N Engl J Med.

[10] Huntley B.J.F., Huntley E.S., Di Mascio D., Chen T., Berghella V., Chauhan S.P. (2020). Rates of maternal and perinatal mortality and vertical transmission in pregnancies complicated by severe acute respiratory syndrome Coronavirus 2 (SARS-Co-V-2) infection: a systematic review. Obstet Gynecol.

[11] Zambrano L.D., Ellington S., Strid P. (2020). Update: characteristics of symptomatic women of reproductive age with laboratory-confirmed SARS-CoV-2 infection by pregnancy status-United States, January 22–October 3, 2020. MMWR Morb Mortal Wkly Rep.

[12] Allotey J., Stallings E., Bonet M. (2020). Clinical manifestations, risk factors, and maternal and perinatal outcomes of coronavirus disease 2019 in pregnancy: living systematic review and meta-analysis. BMJ.

[13] Gonçalves B.M.M., Franco R.P.V., Rodrigues A.S. (2021). Maternal mortality associated with COVID-19 in Brazil in 2020 and 2021: Comparison with non-pregnant women and men. PLoS One.

[14] Metz T.D., Clifton R.G., Hughes B.L. (2022). Association of SARS-CoV-2 infection with serious maternal morbidity and mortality from obstetric complications. JAMA.

[15] Lumbreras-Marquez M.I., Campos-Zamora M., Lizaola-Diaz de Leon H., Farber M.K. (2020). Maternal mortality from COVID-19 in Mexico. Int J Gynaecol Obstet.

[16] Goyal M., Singh P., Singh K., Shekhar S., Agrawal N., Misra S. (2021). The effect of the COVID-19 pandemic on maternal health due to delay in seeking health care: experience from a tertiary center. Int J Gynecol Obstet.

[17] Chmielewska B., Barratt I., Townsend R. (2021). Effects of the COVID-19 pandemic on maternal and perinatal outcomes: a systematic review and meta-analysis. Lancet Glob Health.

[18] Organización Panamericana de la Salud (OPS) (2020).

[19] Khalila A., Kalafata E., Benlioglua C. (2020). SARS-CoV-2 infection in pregnancy: a systematic review and meta-analysis of clinical features and pregnancy outcomes. eClinicalMedicine.

[20] Gajbhiye R.K., Sawant M.S., Kuppusamy P. (2021). Differential impact of COVID-19 in pregnant women from high-income countries and low- to middle-income countries: A systematic review and meta-analysis. Int J Gynaecol Obstet.

[21] Gajbhiye R.K., Mahajan N.N., Waghmare R.B. (2021). Clinical characteristics, outcomes, & mortality in pregnant women with COVID-19 in Maharashtra, India: results from PregCovid registry. Indian J Med Res.

[22] Takemoto M.L.S., Menezes M de O., Nakamura-Pereira M. (2020). The tragedy of COVID-19 in Brazil: 124 maternal deaths and counting. Int J Gynaecol Obstet.

[23] Mendez-Dominguez N, Santos-Zaldívar K, Gomez-Carro S, Datta-Banik S, Carrillo G. Maternal mortality during the COVID-19 pandemic in Mexico: a preliminary analysis during the first year. 10.1186/s12889-021-11325-3 10.1186/s12889-021-11325-3 PMC8253472 34215243

[24] Hantoushzadeh S., Shamshirsaz A.A., Aleyasin A. (2020). Maternal death due to COVID-19. Am J Obstet Gynecol.

[25] Wei S.Q., Bilodeau-Bertrand M., Liu S., Auger N. (2021). The impact of COVID-19 on pregnancy outcomes: a systematic review and meta-analysis. Can Med Assoc J.

[26] Abalos E., Cuesta C., Grosso A.L., Chou D., Say L. (2013). Global and regional estimates of preeclampsia and eclampsia: a systematic review. Eur J Obstet Gynecol Reprod Biol.

[27] Sentilhes L., De Marcillac F., Jouffrieau C. (2020). Coronavirus disease 2019 in pregnancy was associated with maternal morbidity and preterm birth. Am J Obstet Gynecol.

[28] Metz T.D., Clifton R.G., Hughes B.L. (2021). Disease severity and perinatal outcomes of pregnant patients with Coronavirus disease 2019 (COVID-19). Obstet Gynecol.

[29] Papapanou M., Papaioannou M., Petta A. (2021). Maternal and neonatal characteristics and outcomes of covid-19 in pregnancy: an overview of systematic reviews. Int J Environ ResPublic Health.

[30] Osaikhuwuomwan J., Ezeanochie M., Uwagboe C., Ndukwu K., Yusuf S., Ande A. (2021). Clinical characteristics and outcomes for pregnant women diagnosed with COVID-19 disease at the University of Benin Teaching Hospital, Benin City, Nigeria. Pan Afr Med J.

[31] Ata B., Gianaroli L., Lundin K. (2021). Outcomes of SARS-CoV-2 infected pregancies after medically assisted reproduction. Hum Reprod.

[32] La Verde M., Riemma G., Torella M. (2021). Maternal death related to COVID-19: A systematic review and meta-analysis focused on maternal co-morbidities and clinical characteristics. Int J Gynaecol Obstet Off Organ Int Fed Gynaecol Obstet.

[33] Takemoto M.L.S., Menezes M.O., Andreucci C.B. (2020). Maternal mortality and COVID-19. J Matern Fetal Neonatal Med.

[34] Crovetto F., Crispi F., Llurba E. (2021). Impact of severe acute respiratory syndrome coronavirus 2 infection on pregnancy outcomes: a population-based study. Clin Infect Dis.

[35] Vences M.A., Pareja-Ramos J.J., Otero P. (2021). Factors associated with mortality in patients hospitalized with COVID-19: a prospective cohort in a Peruvian national referral hospital. Medwave.

[36] Carle C., Alexander P., Columb M., Johal J. (2013). Design and internal validation of an obstetric early warning score: secondary analysis of the intensive care national audit and research centre case mix programme database. Anaesthesia.

[37] Eman A., Balaban O., Kocayiğit H., Süner K.Ö., Cırdı Y., Erdem A.F. (2021). Maternal and neonatal outcomes of critically ill pregnant and puerperal patients diagnosed with COVID-19 disease: retrospective comparative study. J Korean Med Sci.

[38] Horton R. (2020). Offline: COVID-19 is not a pandemic. Lancet.

[39] Patricia Cifuentes M., Andrea Rodriguez-Villamizar L., Liseth Rojas-Botero M., Arturo Alvarez-Moreno C., Alfredo Fernández-Niño J. (2021). Socioeconomic inequalities associated with mortality for COVID-19 in Colombia: a cohort nationwide study. J Epidemiol Community Health.

[40] Baqui P., Bica I., Marra V., Ercole A., van der Schaar M. (2020). Ethnic and regional variations in hospital mortality from COVID-19 in Brazil: a cross-sectional observational study. Lancet Glob Health.

[41] Serván-Mori E., Seiglie J.A., Gómez-Dantés O., Wirtz V.J. (2022). Hospitalisation and mortality from COVID-19 in Mexican indigenous people: a cross-sectional observational study. J Epidemiol Community Health.

[42] Thaddeus S., Maine D. (1994). Too far to walk: maternal mortality in context. Soc Sci Med.

[43] Vieira R.P. Obstetric observatory BRAZIL-COVID-19: 1031 mater-nal deaths because of COVID-19 and the unequal access to health care services. Available from: https://opendatasus.saude.gov.br/10.6061/clinics/2021/e3120 PMC8221555 34190858

[44] PAHO (2021).

[45] Sachs J., Kroll C., Lafortune G., Fuller G., Woelm F. (2021). Sustainable development report 2021. Sustain Dev Rep.

[46] Maternal Mortality [Internet]. [cited 2022 Mar 31]. Available from: https://www.gatesfoundation.org/goalkeepers/report/2021-report/progress-indicators/maternal-mortality/

[47] The sex, gender and COVID-19 Project | Global Health 50/50 [Internet]. [cited 2022 Mar 29]. Available from: https://globalhealth5050.org/the-sex-gender-and-covid-19-project/

[48] Olivas-Martínez AI, Luis Cá rdenas-Fragoso J, Víctor Jimé nez J, et al. In-hospital mortality from severe COVID-19 in a tertiary care center in Mexico City; causes of death, risk factors and the impact of hospital saturation. 2021; 10.1371/journal.pone.0245772 10.1371/journal.pone.0245772 PMC7857625 33534813

[49] Mathieu E., Ritchie H., Ortiz-Ospina E. (2021). A global database of COVID-19 vaccinations. Nat Hum Behav.

[50] UNICEF/Jake Verzosa. UN News: Low-Income Countries have Received just 0.2 Per cent of all COVID-19 Shots Given UN News. 2021.

[51] Mcclellan M, Udayakumar K, Merson M, Edson G. Reducing global COVID vaccine shortages: new research and recommendations for US leadership. 2021.

[52] UNESCO Office Montevideo and regional bureau for science in Latin America and the Caribbean, Marcela Vélez C. Covid-19 y vacunación en América Latina y el Caribe: desafíos, necesidades y oportunidades. 2021;1–88.

[53] World Health Organization (WHO) (2021). https://www.paho.org/en/documents/xxvi-meeting-pahos-technical-advisory-group-tag-vaccine-preventable-diseases-vaccines.

[54] WHO (2021). https://reliefweb.int/report/world/who-sage-roadmap-prioritizing-uses-covid-19-vaccines-context-limited-supply-approach.

[55] Centers for Disease Control and Prevention (CDC) (2021).

[56] Razzaghi H., Meghani M., Pingali C. (2021). COVID-19 vaccination coverage among pregnant women during pregnancy - eight integrated health care organizations, United States, December 14, 2020–May 8, 2021. MMWR Morb Mortal Wkly Rep.

[57] SAGE (2021).

