# Supplementary material for: Maternal mortality linked to COVID-19 in Latin America: Results from a multi-country collaborative database of 447 deaths
Source: Lancet Reg Health Am. 2022 May 6;12:100269. doi: 10.1016/j.lana.2022.100269 (PMC9073212; doi:10.1016/j.lana.2022.100269)
Supplement: Supplementary file 1 [file mmc1.docx]

**Caption for supplementary material**

**Table 1.** Characteristics of pregnant and puerperium dead women with COVID-19 from Latin America.

**Table 2.** Onset timing, complications, and complications of deaths in pregnancy and puerperium.

**Figure 1.** The onset of symptoms at first consultation (Panel A) or hospital admission (Panel B) for maternal deaths with COVID-19

**Figure 2.** Time from symptoms onset to first consultation, delivery and death in pregnant and postpartum women with COVID-19 in Latin America (n = 447).

**Figure 3.** Organ dysfunction for maternal deaths with COVID-19 in Latin America, according to the WHO classification.

**Appendix 1. STROBE Statement**

**Appendix 2.** Characteristics of pregnant and puerperium dead women with COVID-19 by country
