# Supplementary material for: Maternal mortality linked to COVID-19 in Latin America: Results from a multi-country collaborative database of 447 deaths
Source: Lancet Reg Health Am. 2022 May 6;12:100269. doi: 10.1016/j.lana.2022.100269 (PMC9073212; doi:10.1016/j.lana.2022.100269)
Supplement: Supplementary file 3 [file mmc3.docx]

|  | Honduras  N = 126 | Paraguay  n = 86 | Colombia  n = 84 | Ecuador  n = 55 | Perú  n = 34 | Dominican Republic  n = 30 | Bolivia  n = 21 | Costa Rica  n = 11 |
| --- | --- | --- | --- | --- | --- | --- | --- | --- |
| **Median age** | 29·6 (6·3) | 32·7 (6·4) | 29·0 (6·4) | 30·6 (6·0) | 33·1 (5·5) | 28·5 (4·5) | 30·4 (6·0) | 32·0 (7·8) |
|  | **n/N (%)** | **n/N (%)** | **n/N (%)** | **n/N (%)** | **n/N (%)** | **n/N (%)** | **n/N (%)** | **n/N (%)** |
| **Obstetric history** |  |  |  |  |  |  |  |  |
| Nulliparous | 27/118 (22·8%) | 9/57 (15·7%) | 22/63 (34·9%) | 12/44 (27·2%) | 3/34 (8·8%) | 10/28 (35·7%) | 3/21 (14·3%) | 2/11 (18·2%) |
| 1-2 previous deliveries | 65/118 (55·1%) | 34/57 (59·6%) | 35/63 (55·6%) | 18/44 (40·9%) | 20/34 (58·8%) | 15/28 (53·6%) | 9/21 (42·8%) | 6/11 (54·5%) |
| Multiparous | 26/118 (22·0%) | 14/57 (24·6%) | 6/63 (9·5%) | 14/44 (31·8%) | 11/34 (32·3%) | 3/28 (10·7%) | 9/21 (42·8%) | 3/11 (27·3%) |
| Previous CS | 38/100 (38·0%) | 38/57 (66·7%) | 28/63 (44·4%) | 18/44 (40·9%) | 13/32 (40·6%) | 7/26 (26·9%) | 10/21 (47·6%) | 3/9 (33·3%) |
|  |  |  |  |  |  |  |  |  |
| **Mode of delivery** |  |  |  |  |  |  |  |  |
| Vaginal delivery | 14/85 (16·5%) | 9/47 (19·1%) | 6/46 (13·0%) | 4/37 (10·8%) | 0/20 (0·0%) | 4/22 (18·2%) | 5/17 (29·4%) | 2/8 (25·0%) |
| Cesarean section | 71/85 (83·5%) | 38/47 (80·8%) | 40/46 (86·9%) | 33/37 (89·2%) | 20/20 (100%) | 18/22 (81·8%) | 12/17 (70·6%) | 6/8 (75·0%) |
|  |  |  |  |  |  |  |  |  |
| **Medical history** |  |  |  |  |  |  |  |  |
| Hypertensive disorders: |  |  |  |  |  |  |  |  |
| Chronic Hypertension | 5/123 (4·1%) | 7/33 (21·2%) | 10/81 (12·3%) | 3/54 (5·5%) | 0/34 (0·0%) | 6/29 (20·7%) | 0/20 (0·0%) | 1/6 (16·7%) |
| Preeclampsia | 7/122 (5·7%) | 3/29 (10·3%) | 8/82 (9·7%) | 0/55 (0·0%) | 3/34 (8·8%) | 5/29 (17·2%) | 1/20 (5·0%) | 1/7 (14·3%) |
| Eclampsia | 3/122 (2·4%) | 0/28 (0·0%) | 2/81 (2·5%) | 1/55 (1·8%) | 0/34 (0·0%) | 0/29 (0·0%) | 0/20 (0·0%) | 0/7 (0·0%) |
| Obesity | 27/50 (54·0%) | 9/12 (75·0%) | 19/39 (48·7%) | 6/18 (33·3%) | 11/23 (47%) | 2/7 (28·6%) | 5/13 (38·5%) | 4/6 (66·7%) |
| Diabetes mellitus | 7/122 (5·7%) | 5/32 (15·6%) | 9/80 (11·2%) | 3/55 (5·4%) | 8/34 (23·5%) | 1/29 (3·4%) | 0/20 (0·0%) | 2/8 (25%) |
| Tuberculosis | 0/123 (0%) | 1/28 (3·6%) | 2/81 (2·5%) | 0/55 (0%) | 1/34 (2·9%) | 0/29 (0·0%) | 1/20 (5·0%) | 0/7 (0·0%) |
| HIV | 1/121 (0·8%) | 0/27 (0%) | 2/81 (2·5%) | 3/55 (5·4%) | 0/34 (0%) | 0/26 (0·0%) | 0/20 (0·0%) | 0/7 (0·0%) |
| Asthma | 7/120 (5·8%) | 7/28 (25·9%) | 6/81 (7·4%) | 2/54 (3·7%) | 2/34 (5·9%) | 0/29 (0·0%) | 0/20 (0·0%) | 1/7 (14·3%) |
| Smoking | 0/119 (0·0%) | 0/23 (0·0%) | 1/81 (1·2%) | 0/54 (0·0%) | 0/34 (0·0%) | 0/29 (0·0%) | 0/20 (0·0%) | 0/7 (0·0%) |
| Recreational drug use | 0/119 (0·0%) | 0/23 (0·0%) | 1/81 (1·2%) | 2/54 (3·7%) | 0/34 (0·0%) | 1/29 (3·4%) | 0/20 (0·0%) | 1/8 (12·5%) |
| Alcohol use | 0/119 (0·0%) | 0/23 (0·0%) | 0/81 (0·0%) | 1/53 (1·9%) | 0/34 (0·0%) | 1/29 (3·4%) | 1/20 (5·0%) | 1/8 (12·5%) |

**Appendix 2· Characteristics of pregnant and puerperium dead women with COVID-19 by country**
