# Supplementary material for: Maternal mortality linked to COVID-19 in Latin America: Results from a multi-country collaborative database of 447 deaths
Source: Lancet Reg Health Am. 2022 May 6;12:100269. doi: 10.1016/j.lana.2022.100269 (PMC9073212; doi:10.1016/j.lana.2022.100269)
Supplement: Supplementary file 4 [file mmc4.docx]

**Resumen**

**Antecedentes**: Este estudio tuvo el objetivo de describir las características clínicas de las muertes maternas asociadas a COVID-19 registradas en una base de datos latinoamericana multipaís.

**Métodos**: Se implementó un estudio observacional descriptivo en el que participaron ochos países Latinoamericanos desde el 1^ero^ de marzo 2020 al 29 de noviembre 2021. La información se obtuvo del Sistema Informático Perinatal del Centro Latino Americano de Perinatología, Salud de la Mujer y Reproductiva. Presentamos las variables categóricas como frecuencias y porcentajes y las variables continuas en medianas con rangos inter cuartiles.

**Resultados**: Identificamos un total de 447 muertes. La mediana de edad materna fue de 31 años. 86·4% de las mujeres se infectaron ante del parto, siendo la mayoría de los casos detectados en el tercer trimestre del embarazo (60·3%). Los síntomas más frecuentes en la primera consulta y la admisión fueron disnea (73·0%), fiebre (69·0%), y tos (59·0%). Se reportaron disfunciones orgánicas en 90·4% de las mujeres durante la admisión. Un total de 64·8% de las mujeres fueron ingresadas a cuidados críticos por una mediana de ocho días de estadía. En la mayoría de los casos la muerte ocurrió durante el puerperio, con una media de siete días entre el parto y su ocurrencia. El parto prematuro fue la complicación perinatal más frecuente (76·9%) y 59·9% tuvo bajo peso al nacer.

**Interpretación**: Este estudio describe las características de las muertes maternas durante la pandemia por COVID-19 a partir de una base colaborativa multipaís. Se observaron barreras para el acceso a cuidados intensivos. Los tomadores de decisión deberían trabajar en el fortalecimiento de la conciencia de gravedad, y en estrategias de referencia para evitar potenciales demoras.

**Financiamiento**: Centro Latino Americano de Perinatología, Salud de la Mujer y Reproductiva.
